# Bimodal MRI/Fluorescence Nanoparticle Imaging Contrast Agent Targeting Prostate Cancer

**DOI:** 10.3390/nano14141177

**Published:** 2024-07-10

**Authors:** Hang Xu, Ping Yu, Rajendra P. Bandari, Charles J. Smith, Michael R. Aro, Amolak Singh, Lixin Ma

**Affiliations:** 1 Department of Radiology, University of Missouri, Columbia, MO 65212, USA; 2 Department of Chemical Engineering Graduate Program, University of Missouri, Columbia, MO 65211, USA; 3 Harry S. Truman Veterans’ Memorial Hospital, Columbia, MO 65201, USA; 4 Department of Physics and Astronomy, University of Missouri, Columbia, MO 65211, USA; 5 University of Missouri Research Reactor (MURR), University of Missouri, Columbia, MO 65211, USA

**Keywords:** prostate cancer, bombesin, gastrin-releasing peptide receptor, near-infrared fluorescence imaging, MRI

## Abstract

We developed a novel site-specific bimodal MRI/fluorescence nanoparticle contrast agent targeting gastrin-releasing peptide receptors (GRPrs), which are overexpressed in aggressive prostate cancers. Biocompatible ultra-small superparamagnetic iron oxide (USPIO) nanoparticles were synthesized using glucose and casein coatings, followed by conjugation with a Cy7.5-K-8AOC-BBN [7-14] peptide conjugate. The resulting USPIO(Cy7.5)-BBN nanoparticles were purified by 100 kDa membrane dialysis and fully characterized using transmission electron microscopy (TEM), dynamic light scattering (DLS), Fourier transform infrared (FTIR) spectroscopy, and magnetic resonance imaging (MRI) relaxivity, as well as evaluated for in vitro and in vivo binding specificity and imaging efficacy in PC-3 prostate cancer cells and xenografted tumor-bearing mice. The USPIO(Cy7.5)-BBN nanoparticles had a core diameter of 4.93 ± 0.31 nm and a hydrodynamic diameter of 35.56 ± 0.58 nm. The *r*_2_ relaxivity was measured to be 70.2 ± 2.5 s^−1^ mM^−1^ at 7T MRI. The Cy7.5-K-8AOC-BBN [7-14] peptide-to-nanoparticle ratio was determined to be 21:1. The in vitro GRPr inhibitory binding (IC_50_) value was 2.5 ± 0.7 nM, indicating a very high binding affinity of USPIO(Cy7.5)-BBN to the GRPr on PC-3 cells. In vivo MRI showed significant tumor-to-muscle contrast enhancement in the uptake group at 4 h (31.1 ± 3.4%) and 24 h (25.7 ± 2.1%) post-injection compared to the blocking group (4 h: 15.3 ± 2.0% and 24 h: −2.8 ± 6.8%; *p* < 0.005). In vivo and ex vivo near-infrared fluorescence (NIRF) imaging revealed significantly increased fluorescence in tumors in the uptake group compared to the blocking group. These findings demonstrate the high specificity of bimodal USPIO(Cy7.5)-BBN nanoparticles towards GRPr-expressing PC-3 cells, suggesting their potential for targeted imaging in aggressive prostate cancer.

## 1. Introduction

Prostate cancer is the second leading cause of cancer death among men in the U.S., following lung cancer. In 2024, an estimated 299,010 new cases with 35,250 deaths from prostate cancer are expected [1]. Although the 5-year relative survival rate is nearly 100% for patients diagnosed with localized or regional prostate cancer, it drops to 32% once the cancer metastasizes [1]. Despite advances in cancer diagnosis and treatment, the incidence of advanced-stage prostate cancer has been increasing by 5% per year since 2014 [1]. Therefore, robust diagnostic techniques are critically needed to detect prostate cancer before it metastasizes, which can reduce recurrence rates and improve prognosis. Current techniques, including rectal exams, PSA blood tests, and biopsy, are insufficiently accurate for early detection, often leading to misdiagnosis or over-treatment [2,3]. Consequently, there is an urgent need for a cancer-specific molecular imaging probe with high sensitivity and selectivity for detecting prostate cancer.

Nanotechnology offers new molecular contrast agents that show great potential in earlier and more accurate initial diagnosis, as well as for continuous monitoring of cancer treatments [4,5,6]. The delivery efficiency of the nanoparticles to the tumor is a critical factor in enhancing local imaging contrast. The delivery is controlled by blood circulation, active delivery of the targeting vector to the specific receptors, and passive delivery via the enhanced permeability and retention (EPR) effect [7,8]. A long circulation time is essential for the drug to accumulate at the tumor site, and factors such as coating material and particle size significantly influence blood circulation. Nanomedicine ranging from 10 to 100 nm is particularly suitable for targeting prostate cancer because particles with a hydrodynamic diameter over 100 nm are rapidly cleared by the macrophages of the liver and spleen, while those under 10 nm are rapidly eliminated via the renal system [9,10,11]. Additionally, an advanced coating strategy is needed to enhance the biocompatibility of nanomedicines, reducing uptake by the reticuloendothelial system (RES). Without such a coating, rapid identification by the RES would lead to a rapid capture by the liver, preventing the nanomedicine from accumulating at the targeting site [12].

Superparamagnetic iron oxide nanoparticles (SPIOs) have been widely applied in biomedicine, serving as contrast agents in magnetic resonance imaging (MRI) and magnetic hyperthermia [13,14]. SPIOs offer numerous advantages, including non-toxicity, biodegradability, and versatility for engineering [15,16,17]. In MRI applications, SPIOs enhance contrast by significantly reducing the transverse relaxation time (T_2_) of water protons in absorbing tissues such as tumors, liver, or spleen, and have been used clinically [18]. However, conventional SPIOs face limitations in cancer-related applications due to their relatively large size (hydrodynamic diameter usually over 100 nm) and their tendency to aggregate through mutual magnetic attraction, leading to rapid removal by macrophages in the liver and spleen [11,19]. Moreover, because conventional SPIOs are not specifically directed to disease sites, their local concentration is inadequate for generating significant contrast in MRI images. Recently, ultra-small (5 nm) superparamagnetic iron oxide nanoparticles (USPIOs) have attracted more interest for their ability to enhance T_1_ and T_2_ contrasts in MRI. Strategies have been developed for surface modifications to prolong blood circulation time and incorporate site-specific targeting moieties. Casein, the major component of bovine milk protein, is a biocompatible and degradable biomacromolecule [20]. With both hydrophobic and hydrophilic moieties, casein can be assembled by a cross-link reaction, making it a potential matrix to encapsulate hydrophobic USPIO. This encapsulation can enhance water solubility, biocompatibility, stability, and functionalization of the nanoparticles [20,21].

**Figure 1:**
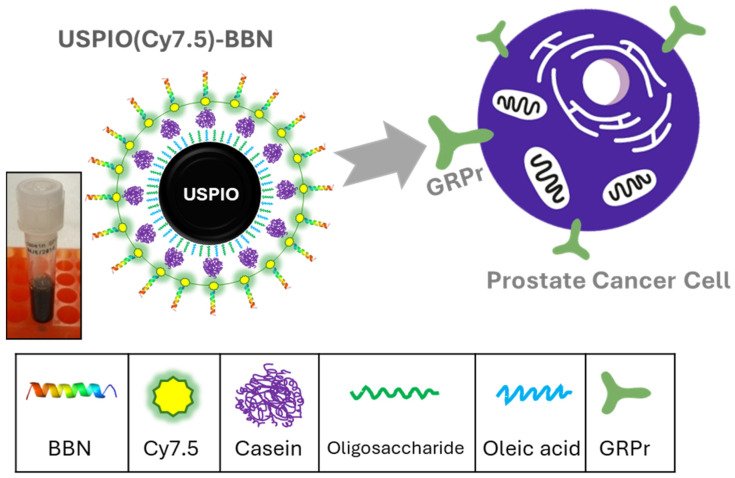
Schematic diagram of interaction between USPIO(Cy7.5)-BBN and prostate cancer cells.

Bombesin (BBN), a 14-amino-acid neuropeptide, has a very high affinity to gastrin-releasing peptide receptors (GRPrs) which are highly expressed in various cancers including prostate cancer, breast cancer, small cell lung cancer, and oral squamous cancer. Over the past decades, significant efforts have been made to synthesize BBN derivative-based molecular imaging probes [22,23,24,25,26,27,28,29,30,31,32,33,34,35,36,37] for specifically targeted molecular imaging and radiotherapy. Among these, several imaging probes, particularly ^68^Ga-BBN derivatives, have been extensively studied in clinical trials, showing positive outcomes of discerning primary prostate and metastasis, as well as demonstrating drug safety [38,39,40,41,42,43,44,45]. In this work, we hypothesize that the BBN derivatives associated with casein-coated USPIO could possess a specific binding ability for targeting prostate cancer with a high affinity. Near-infrared fluorescence imaging (NIRF), a promising candidate for intraoperative imaging and imaging-guided therapy [46,47], features excellent sensitivity, a short operation time, and low costs. The fluorescent signals (600–900 nm) of NIRF imaging probes, such as quantum dots and fluorescent dyes, can penetrate human tissues to a desirable depth. Our previous work regarding bombesin agonist and bombesin antagonist-based NIRF imaging probes demonstrated the potential for delineating prostate tumors from normal tissues with high specificity and binding affinity in pre-clinical studies [32,33,36]. Recognizing the relatively low sensitivity of MRI, we grafted fluorescent dyes to the USPIO to create a bimodal MRI/NIRF imaging probe. This probe integrates MRI’s rich anatomical information and high spatial resolution with NIRF’s high sensitivity. The final compound and its interaction with the GRPr are illustrated in Figure 1.

In this work, the oleic acid-coated USPIO was encapsulated to a casein matrix and loaded with a BBN agonist derivative (K-8AOC-QWAVGHLM-NH_2_) and Cyanine 7.5 (Cy7.5) to form a USPIO(Cy7.5)-BBN conjugate. Several techniques were employed to characterize this compound. Subsequently, a cellular experiment in PC-3 cells and an in vivo study in PC-3 xenografted mouse models were performed to evaluate its in vitro and in vivo binding specificity and affinity to prostate cancer, as well as its MRI/NIRF imaging contrast-enhancing efficacy in small rodent animals.

## 2. Materials and Methods

### 2.1. Materials

All solvents used in this work were either ACS-certified or HPLC-grade. Glucose, casein, glutaraldehyde, 1-ethyl-3-(3-dimethylaminopropyl)carbodiimide (EDAC), and hydroxylamine solution were purchased from Sigma-Aldrich (St. Louis, MO, USA). Dimethylformamide (DMF), sodium bicarbonate, trifluoroacetic acid (TFA), acetonitrile, acetate NHS ester, and sulfo-NHS were obtained from Thermo Fisher Scientific (Waltham, MA, USA). Phosphate buffered saline (PBS) was purchased from Leinco Technologies (St. Louis, MO, USA). Ethanol was obtained from Decon Laboratories (King of Prussia, PA, USA). Iron oxide nanoparticles (5 nm core size, iron concentration 50 mg/2 mL) with an oleic acid coating in chloroform were purchased from Ocean Nanotech (San Diego, CA, USA). Bombesin [1-14] peptides were purchased from American Peptides Co. Inc. (Sunnyvale, CA, USA). ^125^I-Tyr^4^-BBN was obtained from NEN Life Science Inc. (Boston, MA, USA). Cyanine 7.5 NHS ester was purchased from Lumiprobe (Hallandale Beach, FL, USA). Dialysis membranes with a molecular weight cutoff of 100 kDa and 50 kDa were purchased from Spectrum Laboratories Inc. (Rancho Dominguez, CA, USA). All other solvents and reagents were obtained from Sigma-Aldrich (St. Louis, MO, USA) and Thermo Fisher Scientific (Waltham, MA, USA) or otherwise stated, and used as received.

### 2.2. Synthesis and Purification of Cy7.5-K-8AOC-BBN [7-14]NH_2_

**Figure 2:**
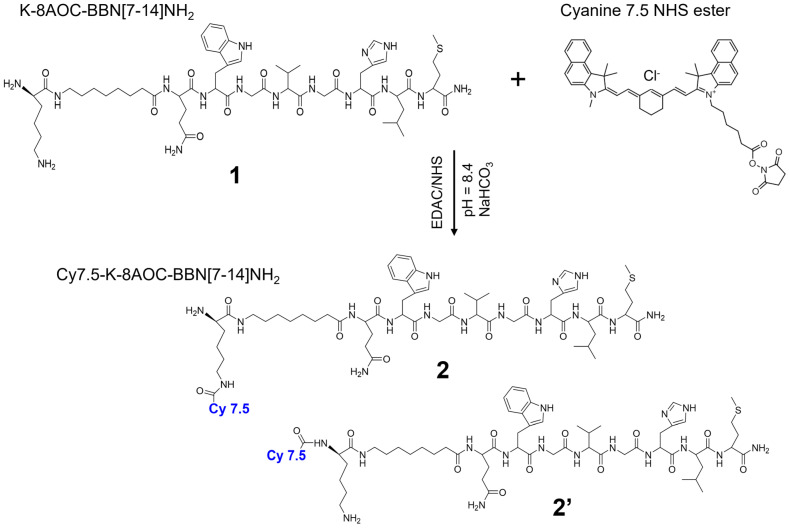
Conjugation of Cyanine 7.5 with **1**, K-8AOC-BBN [7-14]NH_2_ results in two isomers of Cy7.5-K-8AOC-BBN [7-14]NH_2_, **2** and **2′**.

Peptide Lys-8AOC-Gln-Trp-Ala-Val-Gly-His-Leu-NH_2_ (K-8AOC-BBN [7-14]NH_2_, or BBN [7-14] for short) was synthesized using a conventional Fmoc solid-phase peptide synthesizer (SPPS) and purified by high-performance liquid chromatography (HPLC) to a purity greater than 97% in-house or by EZBiolab (Carmel, IN, USA). The conjugation reaction of Cy7.5 to K-8AOC-BBN [7-14]NH_2_ was carried out at a 1:1 molar ratio of Cy7.5: BBN, as depicted in Figure 2. Briefly, approximately 1.2 mg of H_2_N-K-8AOC-BBN [7-14]NH_2_ peptide in 320 µL 0.1 M NaHCO_3_ combined with 100 µL ethanol (pH = 8.3~8.5) was added into 0.768 mg of Cy7.5 NHS ester in 76.8 µL of DMF with stirring. The reaction was performed at 4 °C for 5 h under dark conditions. The crude peptide conjugate was purified using a Shimadzu reverse-phase high-performance liquid chromatography (RP-HPLC) (Kyoto, Japan) system on a specific pre-set gradient (0 min: 95% A and 5% B, 25 min: 30% A and 70% B, 30 min: 5% A and 95% B, where A solvent is 0.1% TFA in pure water and B solvent is 0.1% TFA in acetonitrile), at a 1 mL/min flow rate, and with a UV detection at 280 nm. The product solution was collected and lyophilized on a Savant SpeedVac concentrator. The identity of Cy7.5-K-8AOC-BBN [7-14]NH_2_ was examined on a 4700 MALDITOF/TOF mass spectrometer (Applied Biosystem Inc., Norwalk, CT, USA, now AB Sciex) at the University of Missouri Proteomics Center.

### 2.3. Synthesis and Purification of USPIO(Cy7.5)-BBN

**Figure 3:**
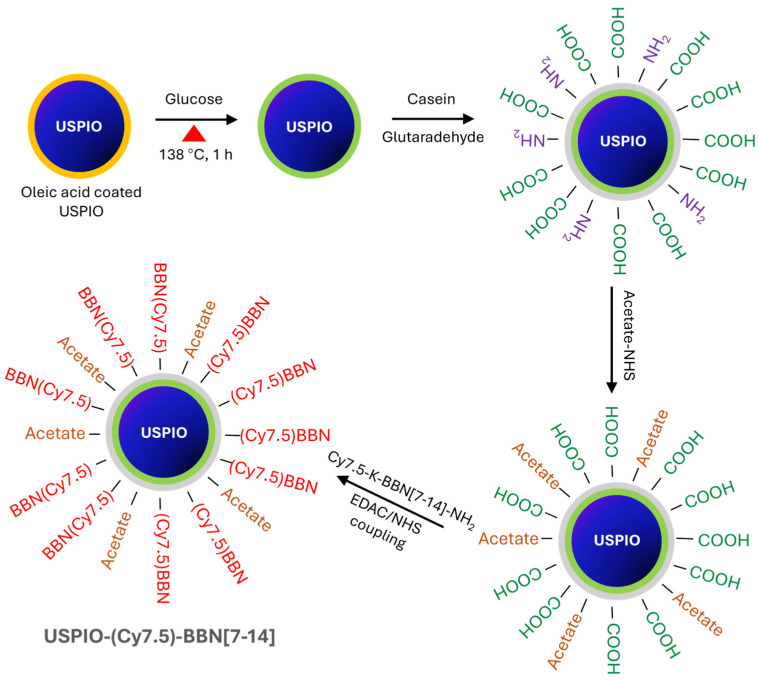
Schematic diagram of synthetic route for USPIO(Cy7.5)-BBN.

As illustrated in Figure 3, the USPIO(Cy7.5)-BBN nanoparticles were derived through a four-step reaction. Steps 1 and 2: surface modification to transfer from water-insoluble to water-soluble USPIO nanoparticle. Step 3: surface amino group blockage and carboxylic acid functionalization of USPIO nanoparticle. Step 4: conjugation of Cy7.5-BBN to USPIO.

Synthesis of glucose-coated USPIO nanoparticle: Glucose (155 mg), in 5.6 mL DMF solution, was preheated and mixed with oleic acid-coated USPIO at a molar ratio of 25,000:1 of glucose to the nanoparticle. The reaction was allowed to proceed for 1 h at 138 °C on a Talboys standard dry block heater (Thorofare, NJ, USA). A 5.8 mL brownish solution was obtained and cooled to room temperature. The mixture was washed three times with ethanol and separated using a SuperMag separator (Ocean Nanotech, San Diego, CA, USA). The oligosaccharide-coated USPIO was obtained and re-dispersed in Milli-Q water.

Synthesis and purification of casein-coated USPIO nanoparticle: Casein was pretreated with 0.01 M NaOH and lyophilized overnight on a Savant SpeedVac concentrator to obtain water-soluble sodium caseinate powders. The oligosaccharide-coated USPIO solution was mixed with the sodium caseinate powder at a 78:1 molar ratio of casein to USPIO and was stirred at room temperature for 4 h. Freshly prepared 0.4% glutaraldehyde was added into this solution (molar ratio of casein to glutaraldehyde at 2:1) dropwise to trigger a cross-linking reaction that was allowed to react for 1 h at room temperature. A dark-brown solution was obtained and purified using a 100 kDa MWCO dialysis membrane to remove any small molecules that were not successfully cross-linked to the nanoparticles. The membrane dialysis was performed three times with gentle stirring in Milli-Q water on a shaker at 4 °C (total time of 48 h).

Surface amino group blockage reaction: The casein-coated USPIO nanoparticles have both amino groups (–NH_2_) and carboxyl groups (–COOH) on the surface. To block the amino groups and create COOH functionalized USPIO nanoparticles, acetate NHS ester was added into the casein-coated USPIO solution at a 60:1 molar ratio of acetate NHS ester to casein. The pH of the reaction mixture was adjusted to 8.15 with NaHCO_3_, and the reaction was carried out at room temperature for 1 h. Finally, the solution was loaded into a 50 kDa MWCO dialysis membrane (Spectrum Inc., Boca Raton, FL, USA) that was suspended in 500 mL Milli-Q water for another membrane dialysis purification for 48 h.

Conjugation of Cy7.5-K-8AOC-BBN [7-14]NH_2_ to USPIO: The final step is the conjugation reaction between the surface-modified hydrophilic USPIO-casein nanoparticle and Cy7.5-K-8AOC-BBN [7-14]NH_2_ to form USPIO(Cy7.5)-BBN nanoparticles. To approximately 0.893 mg (in Fe) COOH functionalized USPIO solution, 2.298 mg EDAC was added followed by the addition of 1.857 mg Sulfo-NHS (molar ratio of EDAC:Sulfo-NHS:NH_2_(reactive group in Cy7.5-K-8AOC-BBN [7-14]NH_2_) = 46:33:1). The reaction mixture was stirred for 15 min and was added to 0.259 µmol Cy7.5-K-8AOC-BBN [7-14]NH_2_ (41.9:1 for BBN to the nanoparticle) in Milli-Q water. The conjugation reaction was allowed for 8 h at 4 °C in the dark. The purification was again carried out by a 50 kDa MWCO membrane dialysis three times under dark conditions and at 4 °C.

### 2.4. Determination of Iron Content of Nanoparticles

The iron concentration in the final USPIO(Cy7.5)-BBN nanoparticles was determined using a Prussian blue staining spectrophotometric method. Standard solutions of different iron concentrations (0.112, 0.0896, 0.0672, 0.0448, 0.0224, 0 mg/mL) were prepared with Feridex (Bayer, Leverkusen, Germany). Each 200 µL solution was added to 200 µL 12.1 N HCl for the acid hydrolysis reaction at 80 °C for 4 h, followed by the addition of 400 µL MilliQ water for dilution and 200 µL 5% Prussian blue for staining. The absorbance of each solution was determined at 690 nm on a Shimazu 1601 UV-vis spectrophotometer (Kyoto, Kyoto Prefecture, Japan). A standard correlation of the light absorbance versus iron concentration was fitted to a linear curve according to Beer’s Law:

(1) $$Absorbance=\log\left(\frac{I_{0}}{I}\right)=\varepsilon Lc$$

where *I*_0_ and *I* are the light intensity before and after passing the solution, *e* is the molar extinction coefficient (or molar absorptivity constant), *L* is the path length of the sample cuvette, and *c* is the concentration of the solution. To prepare the sample solution, 20 µL stock solution was diluted to 200 µL with Milli-Q water and mixed with 200 µL of 12.1 N HCl for 4 h at 80 °C, followed by the addition of 400 µL water and 200 µL 5% Prussian blue solution. The iron concentration was estimated against the Equation (1) fitted standard curve with triplicate measurements.

### 2.5. Determination of Peptide to Nanoparticle Ratio

First, standard solutions of Cy7.5 in 150 µL DMF at different concentrations (0, 6.25, 12.5, 25, 50, 100, and 200 µM) were prepared in a 96-well Cellstar transparent microplate for the absorbance measurement on a Synergy H4 hybrid reader (Biotek, Winooski, VT, USA) at a 788 nm wavelength. The absorbance versus concentration was fitted to a linear curve based on Beer’s Law equation as well. The concentration of Cy7.5-BBN (short for Cy7.5-K-8AOC-BBN [7-14]NH_2_) was estimated against the standard curve of Cy7.5 versus concentration and the molar ratio of 1:1 for BBN to Cy7.5. The iron content of USPIO(Cy7.5)-BBN was obtained by the Prussian blue staining method, and the molar concentration of the USPIO nanoparticles was estimated according to information on iron content to the molar concentration of nanoparticles given by the supplier. The peptide-to-USPIO ratio was then calculated using the molar concentration of Cy7.5-BBN to be divided by the molar concentration of iron oxide nanoparticles.

### 2.6. Fourier Transform Infrared (FTIR) Spectroscopy

To substantiate that glucose and casein have been conjugated on the surface of USPIO, Fourier transform infrared (FTIR) spectroscopy was used to identify chemical bonds in the oleic acid-coated USPIO, glucose-coated USPIO, and casein-coated USPIO. FTIR spectra were acquired and analyzed on a Galaxy series 5000 FTIR spectrometer (Mattson ATI). Each spectrum in the middle infrared range (from 4000 cm^−1^ to 400 cm^−1^) was an average of 16 scans, and the spectral resolution was set to be 2 cm^−1^. The FTIR samples were prepared using a small amount of dry sample (such as glucose, casein, oleic acid-coated USPIO glucose-coated USPIO, or casein-coated USPIO) mixed with dry KBr powders. The mixture was pressed using a press for several tons for 2 min. The background of the spectrum was recorded using a pure KBr pellet.

### 2.7. Transmission Electron Microscopy (TEM)

The morphology and core sizes of casein-coated USPIO and USPIO(Cy7.5)-BBN were studied on a JEOL 1400 transmission electron microscope with an accelerating voltage of 40–120 kV. Each nanoparticle solution was sonicated for 3 min, then 20 µL was dripped on a carbon-coated copper grid for a 5-min incubation. The specimen was air-dried before being inserted into a specimen chamber of the TEM instrument.

### 2.8. Dynamic Light Scattering (DLS)

The hydrodynamic radii of oligosaccharide-coated USPIO, casein-coated USPIO, and USPIO(Cy7.5)-BBN in an aqueous solution were measured using a dynamic light scattering instrument ALV/CGS-3 SLS/DLS system (ALV, Langen, Germany). The tracking of fluctuations of the light intensity, induced by the scattering effect of the nanoparticles’ Brownian Motions in the light path was for 30 s in each run, followed by the analysis by a DLS autocorrelation function for the hydrodynamic radius of the nanoparticles.

### 2.9. MRI Relaxivity Determination

The solutions of USPIO(Cy7.5)-BBN and Feridex (a commercial iron oxide nanoparticle-based MRI T_2_ contrast agent) were prepared in water. R_1_ and R_2_ relaxation rates were determined at iron contents of 0.235, 0.196, 0.157, 0.117, 0.078, and 0.039 mM. The measurements were repeated on two or more independently prepared samples to ensure consistency. A blank sample (0 mM) was also used in the relaxivity measurements for each sample.

Measurements were performed using a 7 Tesla Bruker BioSpec AvanceIII MRI system (Bruker BioSpin, Corporation) equipped with a volume radiofrequency (RF) coil (86 mm inner diameter) at 25 °C. R_1_ and R_2_ were simultaneously measured using a RARE-T1+T2-Map pulse sequence with the slice thickness = 1 mm, matrix = 256 × 128, FOV = 50 × 30 mm, NEX = 1, TE = 11, 22, 33, 44, 55, 66, 77, and 88 ms and TR ranging from 0.482 s to 5 s. The signal intensity of each sample was measured, and the relaxation rate was obtained by the exponential fitting functions in ParaVision 5.1 software (Bruker BioSpin Corporation, Billerica, MA, USA, 2012). The relaxivity *r*_1_ and *r*_2_ values were derived from linear fitting equations of the *R*_1_ and *R*_2_ relaxation rates against the concentrations of the nanoparticle samples, relatively:

(2) $$R_{1}={{(R}_{1})}_{0}+C^{*}r_{1}\;R_{2}={{(R}_{2})}_{0}+C^{*}r_{2}$$

where *C* is the concentration, *R*_1_ and *R*_2_ are the measured relaxation rates of the solution with *R*_1_ *=* 1/*T*_1_ and *R*_2_ *=* 1/*T*_2_, and (*R*_1_*)*_0_ and (*R*_2_*)*_0_ are the baseline relaxation rates when *C* = 0.

### 2.10. Cell Culture and In Vitro Binding Affinity Determination

PC-3 human prostate cancer cells were obtained from the American Type Culture Collection (Manassas, VA, USA) and maintained by the Cell and Immunobiology Core Facility at the University of Missouri. PC-3 cells were grown in a complete growth medium [RPMI1640 media containing 10% heat-inactivated fetal bovine serum (FBS), 1% penicillin from Invitrogen (Carlsbad, CA, USA)] in a Forma water-jacketed incubator (Fisher Scientific) at 37 °C and 5% CO_2_. Cells were grown for 3 days to approximately 90% confluence, detached with Trypsin (Invitrogen, Carlsbad, CA, USA) in 0.25% EDTA solution, washed, and re-suspended in a fresh growth medium.

To determine the binding affinity (inhibitory concentration fifty percent: IC_50_), in vitro competitive cell binding assays for the GRPr were performed against gold standard ^125^I-Tyr^4^-BBN f. Briefly, 3 × 10^4^ PC-3 human prostate cancer cells [RPMI1640 media containing 4.8 mg/mL HEPES and 2 mg/mL BSA (pH7.4)] were incubated at 37 °C for 45 min in the presence of 30,000 cpm ^125^I-Tyr^4^-BBN and increasing concentrations of USPIO(Cy7.5)-BBN. The incubation period was followed by the aspiration of the reaction medium, and the cells were washed three times with ice-cold media. Cell-associated radioactivity was determined by counting in a Wizard 3″ 1480 automatic gamma counter (PerkinElmer, Waltham, MA, USA). IC_50_ values were determined by curve fitting using Prism Software (version 6.0). This procedure was repeated three times for a statistical purpose.

### 2.11. In Vitro Cellular Microscopic Imaging: Uptake, Blocking, Internalization, and Prussian Blue Staining

To determine the binding selectivity of USPIO(Cy7.5)-BBN to GRPr, in vitro cellular imaging was performed in PC-3 cells similar to as previously described [28]. Briefly, for the uptake study, 1 × 10^6^ PC-3 cells were treated with 0.142 nmol USPIO(Cy7.5)-BBN (defined by 0.142 nmol of BBN, equivalent to 0.0067 nmol of the nanoparticle) at 37 °C (5% CO_2_) for 50 min. For the blocking study, 1 × 10^6^ PC-3 cells were pre-incubated with 56.8 nmol native BBN [1-14] for 10 min prior to 50 min incubation with USPIO(Cy7.5)-BBN at 37 °C. The cells were then centrifuged (1000× *g* rcf at 4 °C for 3 min) with the supernatant removed, and the cells were washed once with an ice-cold medium followed by three times with ice-cold PBS. In vitro internalization was performed to assess the degree of USPIO(Cy7.5)-BBN internalized into PC-3 cells. Briefly, PC-3 cells were incubated with 0.142 nmol USPIO(Cy7.5)-BBN at 37 °C (5% CO_2_) for 50 min, followed by an acidic wash with pH = 2.5 buffer (0.2M acetic acid, 0.5M NaCl) for two times and an additional wash with cold PBS. All of the preparation procedures were performed in the dark. The cell-associated fluorescence signals were assessed using an Olympus IX70 (Tokyo, Japan) inverted microscope equipped with bright field, phase and fluorescence optical filters at the MU molecular cytology core. A Cy7 filter set was used with the excitation at 673–748 nm and the emission at 765–835 nm, and images were processed using Metamorph v.7.8.12 software. All of the cell samples were prepared in triplicate.

Prussian blue staining was performed to confirm the coexistence of USPIO and Cy7.5 in PC-3 cells. Briefly, 2 × 10^6^ cells were incubated with 0.284 nmol USPIO(Cy7.5)-BBN for 4 h at 37 °C (5% CO_2_). The treated cells were centrifuged and washed three times with PBS to remove excess USPIO, then fixed with 4% formalin for 30 min or longer, centrifuged again, and washed with Milli-Q water. Cells were dropped onto glass slides and air dried, then covered with a solution of 1% potassium ferrocyanide (50%/50%) in 2% HCl for 20~30 min. The slides were rinsed thoroughly with Milli-Q water and covered with coverslips for microscopic imaging.

### 2.12. Animal Model

In vivo studies were performed in severely compromised immunodeficient (SCID) mice bearing human PC-3 prostate cancer xenografts. Animal studies were conducted in accordance with the highest standards of care as outlined in the NIH’s guide for the care and use of laboratory animals and in accordance with policy and procedures for animal research at the Harry S. Truman Memorial Veterans’ Hospital. Four-to-five-week-old male SCID mice were obtained from Taconic (Germantown, NY, USA). Mice were housed four animals per cage in sterile microisolator cages in a temperature- and humidity-controlled room with a 12-h light/dark schedule. The animals were fed with autoclaved rodent chow (Ralston Purina Company, St. Louis, MO, USA) and provided with water ad libitum. Mice were inoculated with 7 × 10^6^ PC-3 tumor cells in 0.1 mL matri-gel on each right and left flank. Mice were used in the NIRF imaging and MRI experiments between 3–4 weeks post-inoculation of tumor cells. The average body weight of mice was 25–30 g at the time of the study.

### 2.13. In Vivo MRI

MRI studies on tumor-bearing animals were performed on a 7 T Bruker AVANCE III BioSpec MRI scanner (Bruker BioSpin Inc., Billerica, MA, USA) equipped with a gradient insert (400 mT/m, 115 mm I.D.) and a quadrature RF coil (35 mm ID). Mice were anesthetized with 1–2% isoflurane in oxygen via a nose cone over the entire imaging period. A respiratory sensor was placed on the abdomen for respiratory monitoring of vital signs using a Physiological Monitoring System (SA Instruments, Inc., Stony Brook, NY, USA). Body temperature was maintained at 37 °C with warm air circulating in the magnet bore. A rapid acquisition with relaxation enhancement (*RARE*) T_1_-weighted (T1W) imaging sequence was used to obtain coronal images, and a *RARE*-T2 sequence was applied to obtain axial images. The following parameters were applied for the RARE-T1W sequence: TR/TE = 774 ms/9 ms, RARE factor = 4, 16 slices, ST = 1 mm with no gap, matrix = 256 × 256, FOV = 80 × 40 mm (coronal), NEX = 4. For RARE-T2 sequence, TR = 2.5 s, TE = 11, 22, 33, 44, 55, 66, 77, 88 ms, RARE factor = 4, 16 slices, ST = 1 mm with no gap, matrix = 128 × 128, FOV = 30 × 30 mm (axial), and NEX = 4. After the baseline scan, 87 µg (Fe)/mouse (50 µmol Fe/kg) of USPIO(Cy7.5)-BBN in 150 µL isotonic saline was injected intravenously (i.v.) into the mice as a bolus via the tail vein and contrast-enhanced (CE)-MRI images were obtained at 4 h, 24 h, and 48 h after injection. For the blocking study, mice were given an i.v. co-injection of a native BBN [1-14] solution (50 µg/50 µL) and 87 µg (Fe) USPIO(Cy7.5)-BBN in 150 µL isotonic saline, and were imaged at 4 h, 24 h, and 48 h post-injection.

Image analysis and processing were performed using ParaVision 5.1 software (Bruker BioSpin Corporation, 2012). Regions of interest (ROIs) were manually drawn on the tumor, muscle near the tumor, kidney cortex, and liver at each time point. Signal intensity (SI) was measured as the mean of the intensity over the segmented ROI. The contrast-enhancement ratio (*CER*) for an ROI was calculated according to the following equation:

(3) $$CER=\left({CNR}_{post}-{CNR}_{pre}\right)/{CNR}_{pre}\times 100\%$$

where *CNR* is the contrast-to-noise ratio given by *CNR* = (*SI_tumor_ − SI_muscle_*)/*σ_noise_*, and *σ_noise_* is the standard deviation of noise.

### 2.14. NIRF Molecular Imaging

In vivo and ex vivo NIRF imaging were performed on an IVIS Spectrum imaging system (PerkinElmer, Waltham, MA, USA) equipped with a cooled charge-coupled device (CCD) camera and a 150 W quartz halogen light source. The NIRF images were acquired at a filter setting (excitation 745 nm; emission 820 nm) with the following parameters: exposure time (2 s), f/stop (4), binning (M)8, and field of view (13.2 cm). Fluorescence semi-quantification was performed using Living Image 4.4 software (Xenogen, Hopkinton, MA, USA). ROIs were drawn on the tumors and background tissues in the NIRF images and the expression of fluorescence emission intensity was normalized to average radiant efficiency ([p/s/cm^2^/sr]/(µW/cm^2^)).

After the baseline scan, mice were given 87 µg (Fe) of USPIO(Cy7.5)-BBN in 150 µL isotonic saline via tail vein i.v. injection and were imaged at 0.5 h, 1 h, 24 h, and 48 h post-injection. For the blocking study, mice were given i.v. co-injections of a native BBN [1-14] solution (50 µg/50 µL) and 87 µg (Fe) USPIO(Cy7.5)-BBN, and were imaged at 0.5 h, 1 h, 24 h, and 48 h post-injection. After the time point at 48 h post-injection, mice were sacrificed by cervical dislocation under anesthesia (4% isoflurane in 1 L/min O_2_). Organs, including heart, lungs, liver, spleen, kidneys, pancreas, bladder, tumor, muscle, and tibia, were collected, rinsed with PBS, and subjected to an ex vivo NIRF imaging study.

### 2.15. Histopathology

The liver, kidneys, and tumors of the mice from the in vivo study were fixed in 10% neutral-buffered formalin and embedded in paraffin blocks, and slices at 5 mm thickness were made on a microtome. Tissues were stained with Prussian blue (potassium ferrocyanide 10%/HCl 20% (*v*/*v*)) and counterstained with hematoxylin and eosin (H&E) (or nuclear fast red for tumor specimens). The images were taken using a Leica DM5500B microscope (Wetzlar, Germany).

### 2.16. In Vivo Toxicity Assessment

To evaluate the in vivo toxicity of the contrast agent, healthy female CF1 mice (8 weeks old, obtained from Charles River Laboratories) were administered 50 µmol Fe/kg of US-PIO(Cy7.5)-BBN via tail vein injection in 150 µL of isotonic saline. The mice were monitored for body weight and overall health over an extended period. Additionally, T_2_-weighted MRI was performed longitudinally to observe the tissue clearance of US-PIO(Cy7.5)-BBN for up to 35 days.

### 2.17. Statistical Analysis

Quantitative data were expressed as the mean ± standard deviation (SD). Means were compared by analysis of a student’s *t*-test. *p* values of less than 0.05 were considered statistically significant.

## 3. Results and Discussion

In this work, ultra-small iron oxide nanoparticles (5 nm) with an oleic acid coating were encapsulated within a casein matrix. The ultra-small nanoparticles were selected to reduce the potential rapid macrophage capture, although this comes at the expense of MRI relaxivity. Casein, which naturally acts as a nanocarrier for calcium, phosphate, and other biomolecules, is biocompatible and biodegradable [48,49]. It was used to convert the hydrophobic surface to a hydrophilic surface of the nanomedicine in this work. This was followed by the conjugation of Cy7.5-K-8AOC-BBN [7-14]NH_2_ to the nanoparticle platform to enable active targeting of prostate tumors, thereby enhancing delivery efficiency and specificity.

### 3.1. Synthesis and Purifications

Cy7.5 NHS ester was conjugated with the amine on lysine of **1**, K-8AOC-BBN [7-14]NH_2_, resulting in Cy7.5-K-8AOC-BBN [7-14]NH_2_ with two possible isomers, **2** and **2′** (Figure 2). The product of the conjugation reaction was purified by HPLC (Figure S1) and was determined to have a purity of over 95% (Figure S2). The solution corresponding to the third peak was collected and validated to be Cy7.5-K-8AOC-BBN [7-14]NH_2_ by mass spectrometry (Figure S3), with a measured molecular weight (MW) of 1840.004, consistent with 1840.056, the theoretical MW of our desired compound.

**Figure 4:**
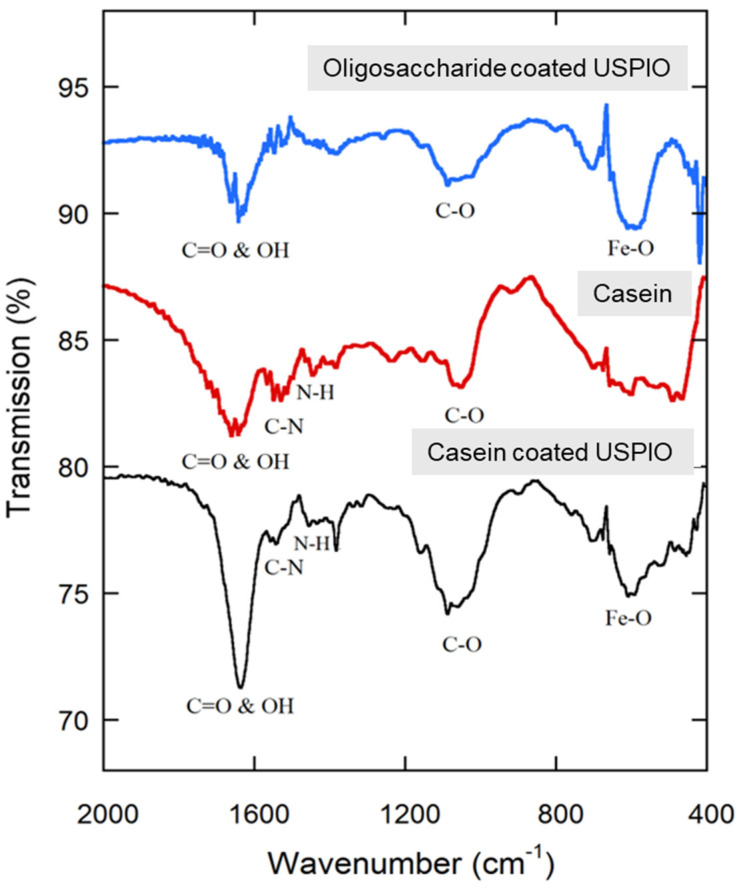
FTIR spectra of oligosaccharide-coated USPIO nanoparticles (blue), pure casein (red), and casein-coated USPIO nanoparticles (black).

Casein-coated USPIO was fabricated via a cross-linking reaction of casein on oleic acid/oligosaccharide-coated USPIO (Figure 3). The presence of the casein coating on the surface of the USPIO was confirmed through FTIR. As shown in Figure 4, the iron oxide stretching bond was found in the spectrum of the oligosaccharide-coated USPIO at 580 cm^−1^. The spectrum of casein displayed a C–N stretching bond at 1533 cm^−1^ and an N–H bending vibration bond at 1455 cm^−1^ as characteristic peaks of peptide bonds. The spectrum of the casein-coated USPIO displayed all three peaks, verifying the formation of the casein coating on the USPIO.

The reaction between the Cy7.5-K-8AOC-BBN [7-14]NH_2_ and the casein-coated USPIO generated a dark brown aqueous solution of USPIO(Cy7.5)-BBN, as shown in the inset of Figure 1. The stability of the USPIO-casein was excellent in that the fluorescence intensity did not significantly decrease, and no visible precipitation was generated over three months.

### 3.2. Morphology, Core Size, Hydrodynamic Diameter, and Peptide-to-Nanoparticle Ratio Determination

**Figure 5:**
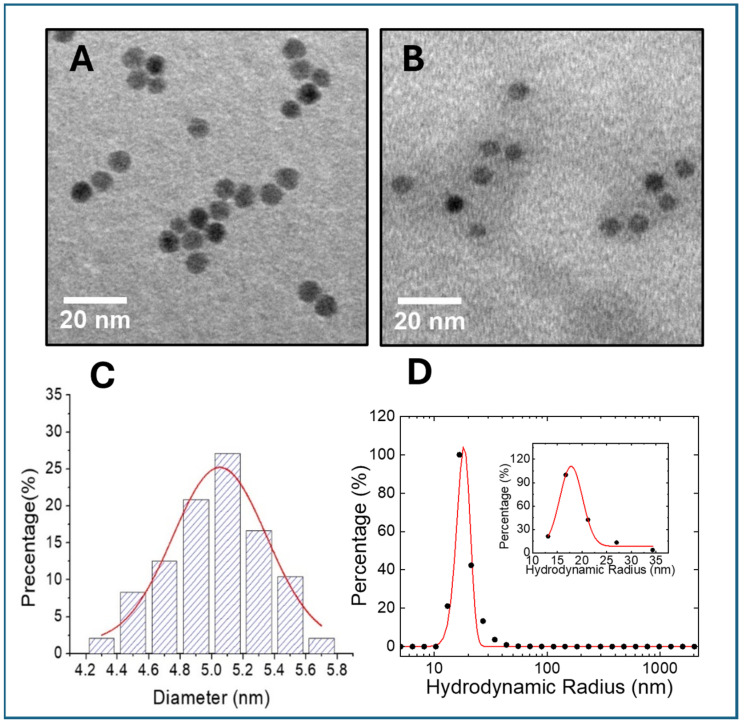
TEM of (**A**) oleic acid/oligosaccharide-coated USPIO and (**B**) USPIO(Cy7.5)-BBN. (**C**) Core diameter histogram of USPIO(Cy7.5)-BBN. (**D**) Number-weighted hydrodynamic radius of USPIO(Cy7.5)-BBN. The radius is displayed on a logarithmic scale. Inset: a fitted curve illustrating the hydrodynamic radius of 17.78 ± 0.29 nm.

The core sizes of the nanoparticles, as shown in the TEM images, remained consistent at all stages (4.77 ± 0.43 nm, 4.79 ± 0.38 nm, and 4.93 ± 0.31 nm for oligosaccharide-coated USPIO, casein-coated USPIO, and USPIO(Cy7.5)-BBN, respectively (Figure 5A–C)). The hydrodynamic diameter was determined to be 35.56 ± 0.58 nm for USPIO(Cy7.5)-BBN (Figure 5D). No larger particles or aggregates were observed in the DLS data. USPIO(Cy7.5)-BBN nanoparticles demonstrated excellent stability due to the unique surface coating strategies employed in this work, which included glucose and casein coating, followed by bioconjugation and a final acylate capping. The resulting hydrodynamic diameter, within the range from 10 nm to 100 nm, is advantageous for nanomedicine applications, as it prolongs blood circulation and escapes from rapid removal by the hepatic/splenic macrophages. The Cy7.5-K-8AOC-BBN [7-14]NH_2_ peptide-to-nanoparticle ratio was determined to be 21:1 through quantification of Cy7.5 fluorescence intensity and the Prussian staining of iron content.

### 3.3. MRI Relaxivity of USPIO(Cy7.5)-BBN

**Figure 6:**
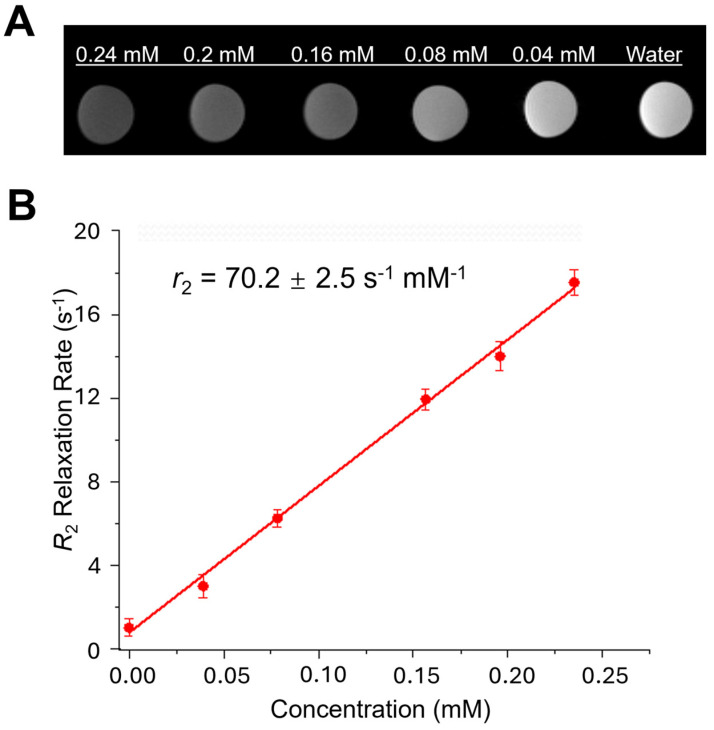
(**A**) T_2_-weighted MRI image at TR of 2500 ms and TE of 55 ms for solutions with different concentrations of USPIO(Cy7.5)-BBN. (**B**) Linear fitting of relaxation rate at different concentrations. The relaxivity was determined to be *r*_2_ = 70.2 ± 2.5 s^−1^ mM^−1^ for USPIO(Cy7.5)-BBN at 7T MRI and room temperature.

A 7T MRI was performed on a series of USPIO(Cy7.5)-BBN solutions with varying iron contents at room temperature to measure relaxivity, which determines the MRI contrast enhancement ability of the contrast agent. The *r*_2_ relaxivity of the USPIO(Cy7.5)-BBN was determined to be 70.2 ± 2.5 s^−1^ mM^−1^, through linear regression curve fitting of the measured *R*_2_ relaxation rates against various concentrations (Figure 6). The *r*_1_ relaxivity of the USPIO(Cy7.5)-BBN was determined to be 1.830 ± 0.225 s^−1^ mM^−1^ (Figure S4). With a similar metal core size of 5 nm, the clinical contrast agent Feridex had a dextran surface coating and a hydrodynamic size of 160 nm [50]. The *r*_1_ and *r*_2_ relaxivities of Feridex were measured to be 0.892 ± 0.002 s^−1^ mM^−1^ and 144.7 ± 0.9 s^−1^ mM^−1^, respectively, at 7T MRI and room temperature. The higher *r*_2_ and lower *r*_1_ of Feridex are attributed to its large hydrodynamic size [50]. Compared to Feridex, the *r*_2_*/r*_1_ ratio of USPIO(Cy7.5)-BBN was 39, an improvement over Feridex’s ratio of 162. Another clinically used contrast agent, Ferumoxtran (Combidex), had a metal core diameter of 5.85 nm and a hydrodynamic diameter of 35 nm, with an *r*_2_ relaxivity of 65 s^−1^ mM^−1^ at 1.5 T [50]. The relaxivity value of USPIO(Cy7.5)-BBN indicates that it is a highly competitive T_2_ contrast agent for nanoparticles of this core size.

### 3.4. Binding Affinity of USPIO(Cy7.5)-BBN to Prostate Cancer Cells

**Figure 7:**
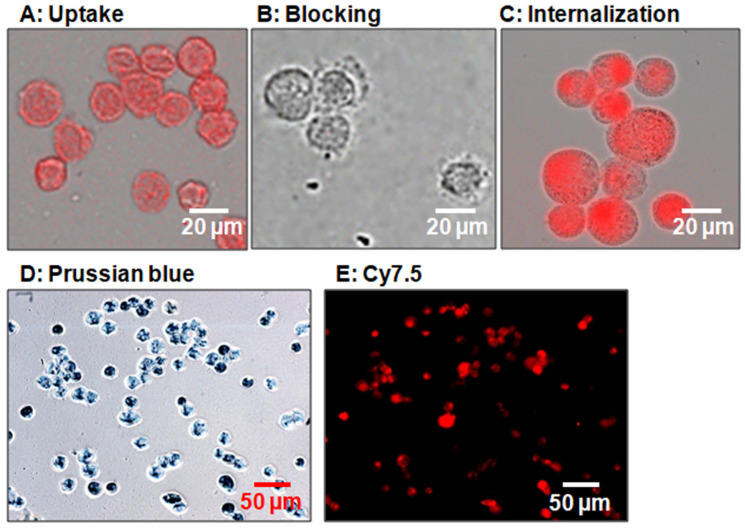
Microscopic cellular images. (**A**) Uptake: PC-3 cells treated with USPIO(Cy7.5)-BBN. (**B**) Blocking: PC-3 cells pre-treated with 400-fold BBN [1-14] followed by incubation with USPIO(Cy7.5)-BBN. (**C**) Internalization: PC-3 cells treated with USPIO(Cy7.5)-BBN and further washed with pH = 2.5 buffer. Prussian blue staining of PC-3 cells treated with USPIO(Cy7.5)-BBN confirmed the co-existence of iron elements (dark blue) (**D**) and Cy7.5 fluorescence signals (red) (**E**) bound to PC-3 cells.

**Figure 8:**
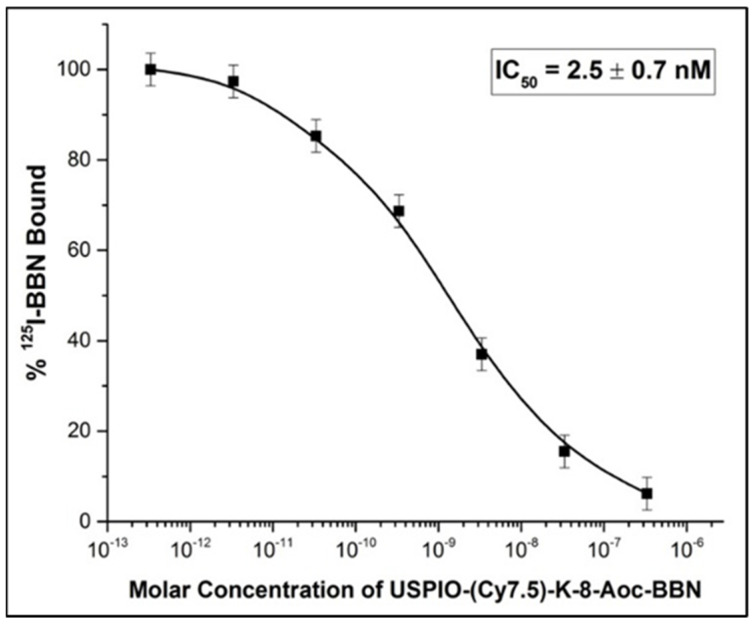
IC_50_ determination of USPIO(Cy7.5)-BBN against 125I-Tyr4-BBN in PC-3 cells.

In vitro results revealed that USPIO(Cy7.5)-BBN binds to PC-3 prostate cancer cells with a high uptake and high specificity to GRP receptors (Figure 7). Fluorescence microscopic imaging showed strong Cy7.5 fluorescence in PC-3 cells treated with USPIO(Cy7.5)-BBN (uptake group, Figure 7A). This binding was significantly inhibited in the presence of unlabeled BBN [1-14] (blocking group, Figure 7B). The internalization study showed that strong Cy7.5 fluorescence remained after an acidic wash, indicating the internalization of this compound by PC-3 cells (Figure 7C). Further experiments with Prussian blue staining confirmed the coexistence of iron oxide nanoparticles with Cy7.5 in PC-3 cells, with the red representing the fluorescence of Cy7.5 and the dark blue representing Prussian blue from iron elements (Figure 7D,E). The binding affinity of USPIO(Cy7.5)-BBN was quantitatively evaluated using a ^125^I-Tyr^4^-BBN competitive binding assay. The IC_50_ value was determined to be 2.5 ± 0.7 nM for USPIO(Cy7.5)-BBN, indicating a very high bind affinity for the GRPr on PC-3 cells (Figure 8).

### 3.5. In Vivo NIRF & MRI Molecular Imaging

**Figure 9:**
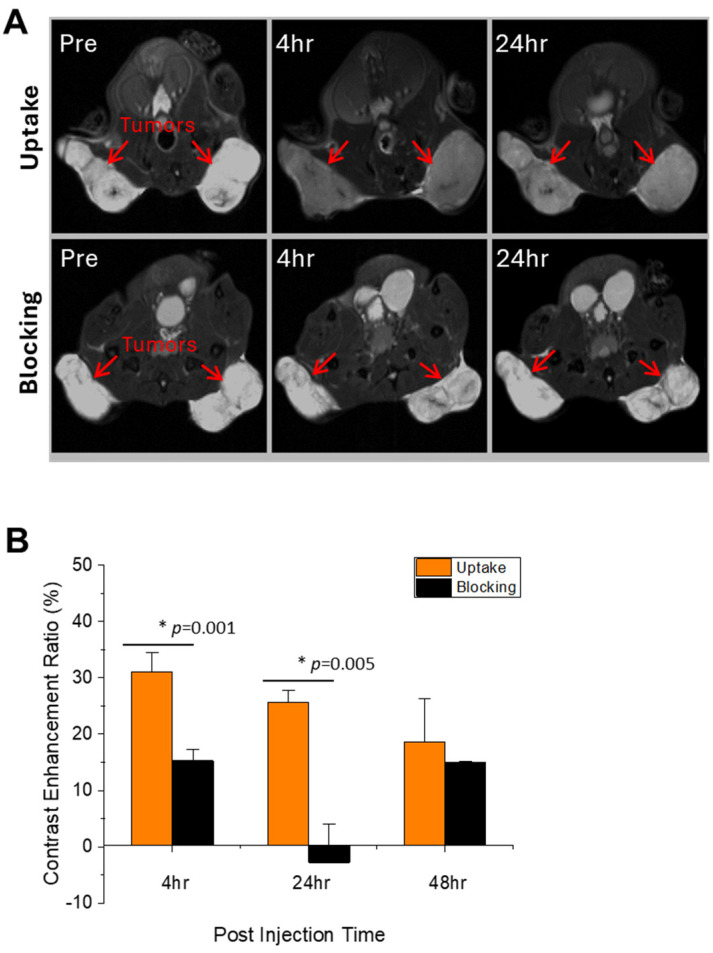
In vivo 7T MRI of male SCID mice bearing bilateral flank PC-3 tumors pre- and post-intravenous injections of USPIO(Cy7.5)-BBN. (**A**) Representative T_2_-weighted MRI of the uptake group (upper panel) compared to the blocking group (lower panel). (**B**) The tumor contrast-enhancement ratio (CER) in the uptake and blocking groups at 4 h, 24 h, and 48 h post-injection. Red arrows indicate tumors. Asterisks (*) denote statistical significance.

The in vivo specific binding efficacy of USPIO(Cy7.5)-BBN was evaluated in a xenografted PC-3 model using NIRF imaging and MRI. For the blocking group, BBN [1-14] and the USPIO(Cy7.5)-BBN were co-injected into the PC-3 xenograft model, with the assumption that the smaller BBN [1-14] would reach tumor sites faster than the BBN-conjugated nanoparticles, facilitating prolonged NIRF and MRI imaging. In vivo MRI images were acquired at 4 h, 24 h, and 48 h post-injection, showing a visible increase in the tumor-to-muscle contrast following the injection of USPIO(Cy7.5)-BBN in the uptake group (Figure 9A). As shown in Figure 9B, the tumor CER of the uptake group was significantly higher than the tumor CER of the blocking group at 4 h (31.1 ± 3.4% versus 15.3 ± 2.0%; *p* = 0.001) and 24 h (25.7 ± 2.1% versus −2.8 ± 6.8%; *p* = 0.005) post-injection, demonstrating the GRPr-specific MRI contrast enhancement by USPIO(Cy7.5)-BBN.

**Figure 10:**
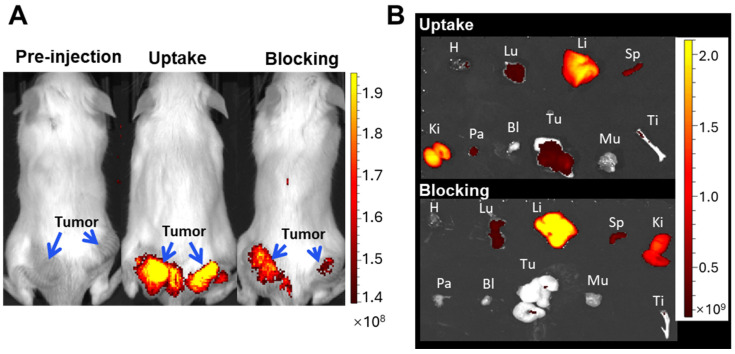
(**A**) In vivo NIRF imaging (IVIS Spectrum, Ex: 745 nm/Em: 800 nm) of SCID mice bearing PC-3 tumors pre- and 24 h post-injection of USPIO(Cy7.5)-BBN via the tail vein in the uptake group as compared to the blocking group. (**B**) Ex vivo NIRF imaging of organs collected 48 h post-injection in the uptake group as compared to the blocking group. H, heart; Lu, lung; Li, liver; Sp, spleen; Ki, kidney; Pa, pancreas; Bl, bladder; Tu, tumor; Mu, muscle; Ti, tibia.

The in vivo NIRF study was conducted in parallel with the in vivo MRI study. The tumors displayed more intense fluorescence signals in the uptake group than in the blocking group (Figure 10A). Due to factors such as the distance of the mouse from the transducer and light scattering during tissue penetration that impacts fluorescence measurements in the in vivo NIRF imaging test, the mice were sacrificed and dissected for a quantitative ex vivo evaluation at 48 h post-injection, as shown in Figure 10B, and the bio-distribution data are in Figure S5. The tumor-to-muscle ratio was determined to be 4.35 in the uptake group, which is higher than the 2.86 shown in the blocking group. The pancreas also showed a significantly higher fluorescent signal intensity in the uptake group as compared to that in the blocking group, which is attributed to the high expression of GRPr in this tissue. These data quantitatively demonstrate that USPIO(Cy7.5)-BBN has a high binding affinity and specificity for prostate tumors and pancreas through specific GRPr targeting.

Moreover, the significantly darkened MRI signal in the tumor site, concurrent with the elevated signal intensity of the same tissues in the NIRF images, confirms the co-existence of Cy7.5 and USPIO at the tumor site, proving the integrity of this drug in the process of delivery. An interesting phenomenon worth noting is that the relative NIRF signal contrast between liver and kidney was significantly altered when BBN [1-14] was co-injected with the nanoparticles in the blocking group. The liver signal intensity in the blocking group was higher than that in the uptake group, and, correspondingly, the kidney signal intensity was lower in the blocking group than in the uptake group. This observation is consistent with our previously published work on bombesin antagonist-based NIRF imaging probes [32], indicating a change in the excretion route with the co-injection of BBN [1-14]. This may be due to the antidiuretic effect of BBN reported in previous research [49], but a detailed study is needed to address this issue.

### 3.6. Histopathology

**Figure 11:**
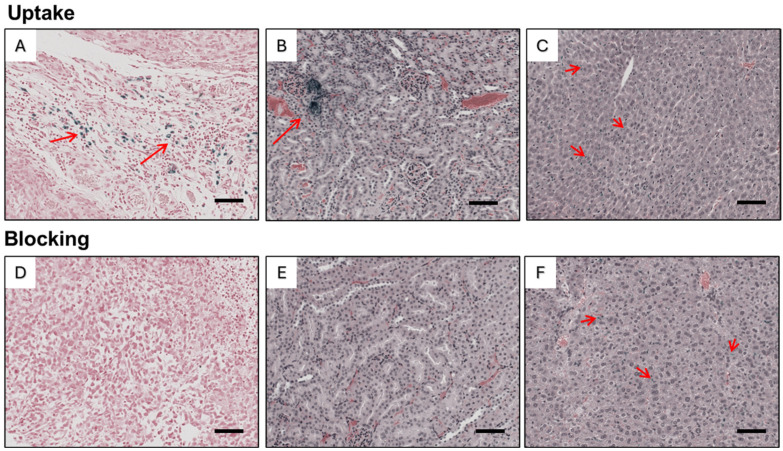
Histopathological imaging of Prussian blue staining and H&E sections of tumor (**A**,**D**), kidney (**B**,**E**), and liver (**C**,**F**) in the uptake and blocking group. Arrows indicate the USPIO(Cy7.5)-BBN nanoparticles with Prussian blue staining. The scale bar represents 100 µm.

The distribution patterns of iron oxide nanoparticles in the tumors, livers, and kidneys of mice from the in vivo study were examined. The sections of livers and kidneys were dual-stained with Prussian blue and H&E, while the tumor sections were dual-stained with Prussian blue and nuclear fast red. As shown in Figure 11A, the distribution of USPIO(Cy7.5)-BBN was not uniform in tumors, implying heterogeneity in the nanoparticles’ access to the cancer cells within the solid tumor. USPIO(Cy7.5)-BBN was observed in the tumors of the uptake group; however, the level was much lower in the blocking group (Figure 11D), suggesting that the delivery to the tumor can be inhibited by blocking the GRPr binding sites with BBN [1-14]. As shown in Figure 11B, the USPIO(Cy7.5)-BBN is distributed unevenly in kidneys. Regions with concentrated USPIO were observed in the kidneys from the uptake group, but rarely in the kidneys from the blocking group (Figure 11E), suggesting a lower quantity of USPIO removed through renal excretion in the blocking group. This finding is consistent with the results of the ex vivo NIRF study. USPIO(Cy7.5)-BBN exists in the liver, but dense spots of USPIO were hardly observed (Figure 11C,F), indicating a widespread and even distribution pattern of USPIO(Cy7.5)-BBN in the liver.

### 3.7. In Vivo Toxicity Assessment

**Figure 12:**
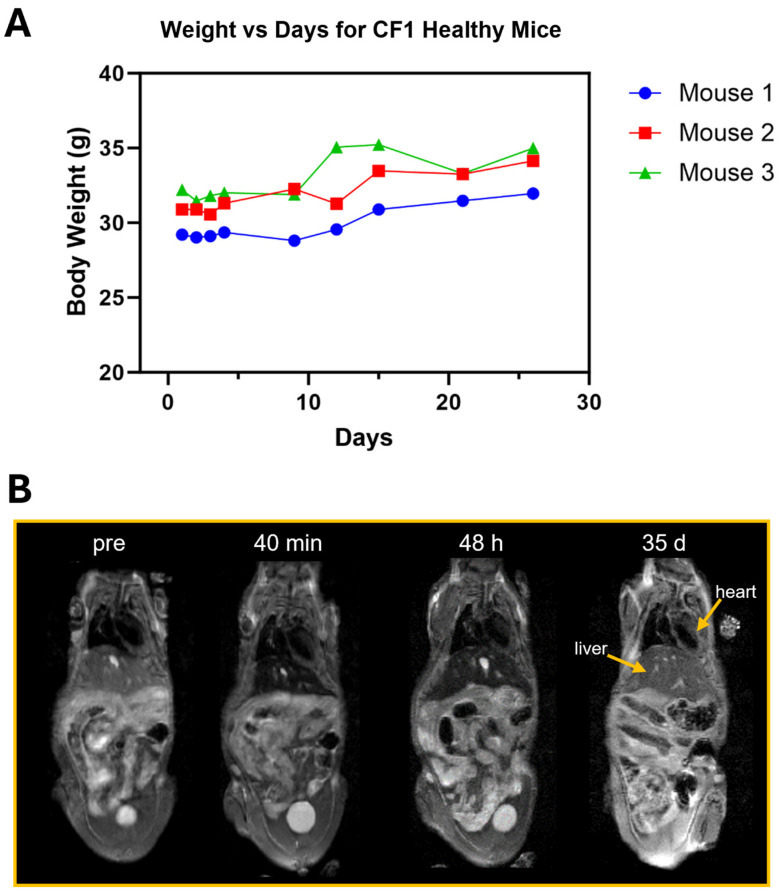
Intravenous administration of 50 µmol Fe/kg of USPIO(Cy7.5)-BBN in 150 µL to healthy CF1 mice did not show toxicity over an extended period. (**A**) The body weights showed normal increases (*n* = 3). (**B**) T_2_-weighted MRI showed that the uptake of the contrast agent in the liver (indicated by dark enhancement at 40 min and 48 h post-injection) was cleared by 35 days post-injection.

No toxicity was observed in healthy female CF1 mice administered 50 µmol Fe/kg of US-PIO(Cy7.5)-BBN via tail vein injection and monitored for overall health over an extended period. All mice exhibited normal health status and normal body weight increases over the course of one month (Figure 12A). T_2_-weighted MRI showed that the contrast agent was primarily distributed in the liver tissues from 40 min to 48 h post-injection, as indicated by the intense contrast enhancement in the liver (Figure 12B). The liver enhancement gradually decreased over time and was cleared by 35 days post-injection (Figure 12B).

Up until now, the long-term toxicity of iron oxide nanoparticles has not been documented [51]. Administrated USPIO is often found largely in the liver and spleen due to the rich presence of macrophages in these organs. Once inside the cells through endocytosis, iron oxide nanoparticles are decomposed into iron elements in endosomes and lysosomes where an acidic environment is present. Finally, these free iron elements will be merged into a cellular iron pool and utilized for the generation of hemoglobin [52,53]. However, a formation of excess reactive oxygen species (ROS) may occur in the case of cells that are overexposed to USPIO, and that may result in apoptosis, or cell death, by disrupting normal cellular functions [9,52]. Therefore, reducing the dosage through specific targeted delivery of USPIO is necessary for preventing negative physiological responses. During our in vivo studies, mice with intravenous administrations of USPIO(Cy7.5)-BBN behaved normally, and their body weight remained stable for over one month for three healthy mice, preliminarily confirming no toxicity of this compound. USPIO(Cy7.5)-BBN can be loaded with radioactive tracers to leverage their extraordinary sensitivity in imaging, providing more detailed information regarding bio-distribution, pharmacokinetics, and excretion. This nanoparticle platform can also be exploited to incorporate therapeutic agents for tumor theranostics, where therapy can be real-time monitored and evaluated using MRI/NIRF multi-modality imaging. Although diverse new therapeutic strategies against malignant tumors have emerged in recent years, along with improvements in conventional treatments [54,55,56], there is still a long way to go in significantly reducing cancer-related mortality. Therefore, early detection is crucial to win the battle against cancer.

## 4. Conclusions

In conclusion, we developed a GRPr-specific bimodal MRI/fluorescence nanoparticle contrast agent for prostate cancer imaging. This study investigated the surface coating and functionalization of ultra-small iron oxide nanoparticles, and the bioconjugation of USPIO(Cy7.5)-BBN. The resulting USPIO(Cy7.5)-BBN nanoparticles demonstrated high specificity towards GRPr-expressing PC-3 cells, indicating their potential for targeted imaging of aggressive prostate cancer. In an in vitro test with PC-3 cells, an internalized GRPr-specific cell binding with a high binding affinity of IC_50_ = 2.5 ± 0.7 nM for PC-3 cells was verified. In an in vivo study with a prostate tumor-bearing SCID mouse model, the compound was demonstrated to significantly enhance the contrast of prostate cancer from the immediate nearby tissues in NIRF/MRI multi-modality imaging, via specific binding of bombesin to the GRP receptors. The outstanding multi-modality imaging capability of this nanoparticle platform, made of biocompatible materials, promises a robust imaging tool for improving prostate cancer diagnosis and image-guided therapy in the clinic.

## Data Availability

The original contributions presented in the study are included in the article/Supplementary Material, further inquiries can be directed to the corresponding author/s.

## Supplementary Materials

The following supporting information can be downloaded at: https://www.mdpi.com/article/10.3390/nano14141177/s1, Figures S1–S3: HPLC, purity, and mass spectrum of Cy7.5-K-8AOC-BBN[7-14]NH_2_; Figure S4: *r*_1_ relaxivity of USPIO(Cy7.5)-BBN; Figure S5: bio-distribution of USPIO(Cy7.5)-BBN in SCID mice bearing PC-3 tumors.

## Abbreviations

BBN, bombesin; GPR, gastrin-releasing peptide; GPRr, gastrin-releasing peptide receptor; Cy7.5, Cyanine 7.5; SPIO, superparamagnetic iron oxide nanoparticles; USPIO, ultra-small superparamagnetic iron oxide nanoparticles; TMR, tumor-to-muscle ratio.

## References

[1] Siegel R.L., Giaquinto A.N., Jemal A. (2024). Cancer statistics, 2024. CA A Cancer J. Clin..

[2] Howrey B., Kuo Y.-F., Lin Y.-L., Goodwin J.S. (2013). The impact of PSA screening on prostate cancer mortality and overdiagnosis of prostate cancer in the United States. J. Gerontol. Ser. A Biol. Sci. Med. Sci..

[3] Schröder F.H., Hugosson J., Roobol M.J., Tammela T.L., Ciatto S., Nelen V., Kwiatkowski M., Lujan M., Lilja H., Zappa M. (2012). Prostate-cancer mortality at 11 years of follow-up. N. Engl. J. Med..

[4] Choi Y.E., Kwak J.W., Park J.W. (2010). Nanotechnology for early cancer detection. Sensors.

[5] Kircher M.F., de la Zerda A., Jokerst J.V., Zavaleta C.L., Kempen P.J., Mittra E., Pitter K., Huang R., Campos C., Habte F. (2012). A brain tumor molecular imaging strategy using a new triple-modality MRI-photoacoustic-Raman nanoparticle. Nat. Med..

[6] Phillips E., Penate-Medina O., Zanzonico P.B., Carvajal R.D., Mohan P., Ye Y., Humm J., Gönen M., Kalaigian H., Schöder H. (2014). Clinical translation of an ultrasmall inorganic optical-PET imaging nanoparticle probe. Sci. Transl. Med..

[7] Maeda H., Wu J., Sawa T., Matsumura Y., Hori K. (2000). Tumor vascular permeability and the EPR effect in macromolecular therapeutics: A review. J. Control. Release.

[8] Tanaka T., Shiramoto S., Miyashita M., Fujishima Y., Kaneo Y. (2004). Tumor targeting based on the effect of enhanced permeability and retention (EPR) and the mechanism of receptor-mediated endocytosis (RME). Int. J. Pharm..

[9] Thomas R.G., Moon M.J., Lee H., Sasikala A.R.K., Kim C.S., Park I.-K., Jeong Y.Y. (2015). Hyaluronic acid conjugated superparamagnetic iron oxide nanoparticle for cancer diagnosis and hyperthermia therapy. Carbohydr. Polym..

[10] Weissleder R., Elizondo G., Wittenberg J., Rabito C.A., Bengele H.H., Josephson L. (1990). Ultrasmall superparamagnetic iron oxide: Characterization of a new class of contrast agents for MR imaging. Radiology.

[11] Xie J., Liu G., Eden H.S., Ai H., Chen X. (2011). Surface-engineered magnetic nanoparticle platforms for cancer imaging and therapy. Acc. Chem. Res..

[12] Rao L., Bu L.-L., Xu J.-H., Cai B., Yu G.-T., Yu X., He Z., Huang Q., Li A., Guo S.-S. (2015). Red Blood Cell Membrane as a Biomimetic Nanocoating for Prolonged Circulation Time and Reduced Accelerated Blood Clearance. Small.

[13] Bañobre-López M., Teijeiro A., Rivas J. (2013). Magnetic nanoparticle-based hyperthermia for cancer treatment. Rep. Pract. Oncol. Radiother..

[14] Pouliquen D., Le Jeune J.J., Perdrisot R., Ermias A., Jallet P. (1991). Iron oxide nanoparticles for use as an MRI contrast agent: Pharmacokinetics and metabolism. Magn. Reson. Imaging.

[15] Liong M., Lu J., Kovochich M., Xia T., Ruehm S.G., Nel A.E., Tamanoi F., Zink J.I. (2008). Multifunctional inorganic nanoparticles for imaging, targeting, and drug delivery. ACS Nano.

[16] Doane T.L., Burda C. (2012). The unique role of nanoparticles in nanomedicine: Imaging, drug delivery and therapy. Chem. Soc. Rev..

[17] Ma X., Zhao Y., Liang X.J. (2011). Theranostic nanoparticles engineered for clinic and pharmaceutics. Acc. Chem. Res..

[18] Nakamura H., Ito N., Kotake F., Mizokami Y., Matsuoka T. (2000). Tumor-detecting capacity and clinical usefulness of SPIO-MRI in patients with hepatocellular carcinoma. J. Gastroenterol..

[19] Weissleder R., Hahn P.F., Stark D.D., Elizondo G., Saini S., Todd L.E., Wittenberg J., Ferrucci J.T. (1988). Superparamagnetic iron oxide: Enhanced detection of focal splenic tumors with MR imaging. Radiology.

[20] Elzoghby A.O., El-Fotoh W.S., Elgindy N.A. (2011). Casein-based formulations as promising controlled release drug delivery systems. J. Control Release.

[21] Huang J., Wang L., Lin R., Wang A.Y., Yang L., Kuang M., Qian W., Mao H. (2013). Casein-coated iron oxide nanoparticles for high MRI contrast enhancement and efficient cell targeting. ACS Appl. Mater. Interfaces.

[22] Biddlecombe G.B., Rogers B.E., De Visser M., Parry J.J., De Jong M., Erion J.L., Lewis J.S. (2007). Molecular imaging of gastrin-releasing peptide receptor-positive tumors in mice using ^64^Cu- and ^86^Y-DOTA-(Pro1,Tyr 4)-bombesin(1-14). Bioconjugate Chem..

[23] Hoffman T.J., Gali H., Smith C.J., Sieckman G.L., Hayes D.L., Owen N.K., Volkert W.A. (2003). Novel series of ^111^In-labeled bombesin analogs as potential radiopharmaceuticals for specific targeting of gastrin-releasing peptide receptors expressed on human prostate cancer cells. J. Nucl. Med..

[24] Liu Z., Li Z.B., Cao Q., Liu S., Wang F., Chen X. (2009). Small-animal PET of tumors with ^64^Cu-labeled RGD-bombesin heterodimer. J. Nucl. Med..

[25] Liu Z., Yan Y., Chin F.T., Wang F., Chen X. (2009). Dual integrin and gastrin-releasing peptide receptor targeted tumor imaging using ^18^F-Labeled PEGylated RGD-bombesin heterodimer ^18^F-FB-PEG3-Glu-RGD-BBN. J. Med. Chem..

[26] Mansi R., Wang X., Forrer F., Waser B., Cescato R., Graham K., Borkowski S., Reubi J.C., Maecke H.R. (2011). Development of a potent DOTA-conjugated bombesin antagonist for targeting GRPr-positive tumours. Eur. J. Nucl. Med. Mol. Imaging.

[27] Pinski J., Halmos G., Yano T., Szepeshazi K., Qin Y., Ertl T., Schally A.V. (1994). Inhibition of growth of MKN45 human gastric-carcinoma xenografts in nude mice by treatment with bombesin/gastrin-releasing-peptide antagonist (RC-3095) and somatostatin analogue RC-160. Int. J. Cancer.

[28] Rogers B.E., Bigott H.M., McCarthy D.W., Della Manna D., Kim J., Sharp T.L., Welch M.J. (2003). MicroPET imaging of a gastrin-releasing peptide receptor-positive tumor in a mouse model of human prostate cancer using a ^64^Cu-labeled bombesin analogue. Bioconjugate Chem..

[29] Rogers B.E., Manna D.D., Safavy A. (2004). In Vitro and In Vivo Evaluation of a ^64^Cu-Labeled Polyethylene Glycol-Bombesin Conjugate. Cancer Biother. Radio..

[30] Stott Reynolds T.J., Schehr R., Liu D., Xu J., Miao Y., Hoffman T.J., Rold T.L., Lewis M.R., Smith C.J. (2015). Characterization and evaluation of DOTA-conjugated Bombesin/RGD-antagonists for prostate cancer tumor imaging and therapy. Nucl. Med. Biol..

[31] Sun B., Schally A.V., Halmos G. (2000). The presence of receptors for bombesin/GRP and mRNA for three receptor subtypes in human ovarian epithelial cancers. Regul. Pept..

[32] Xu H., Bandari R.P., Lee L., Li R., Yu P., Smith C.J., Ma L. (2018). Design, Synthesis, and in Vitro and in Vivo Evaluation of High Affinity and Specificity Near-Infrared Fluorescent Bombesin Antagonists for Tumor Imaging. J. Med. Chem..

[33] Cai Q.Y., Yu P., Besch-Williford C., Smith C.J., Sieckman G.L., Hoffman T.J., Ma L. (2013). Near-infrared fluorescence imaging of gastrin releasing peptide receptor targeting in prostate cancer lymph node metastases. Prostate.

[34] Li R., Gao R., Wang Y., Liu Z., Xu H., Duan A., Zhang F., Ma L. (2020). Gastrin releasing peptide receptor targeted nano-graphene oxide for near-infrared fluorescence imaging of oral squamous cell carcinoma. Sci. Rep..

[35] Li R., Gao R., Zhao Y., Zhang F., Wang X., Li B., Wang L., Ma L., Du J. (2022). pH-responsive graphene oxide loaded with targeted peptide and anticancer drug for OSCC therapy. Front. Oncol..

[36] Ma L., Yu P., Veerendra B., Rold T.L., Retzloff L., Prasanphanich A., Sieckman G., Hoffman T.J., Volkert W.A., Smith C.J. (2007). In vitro and in vivo evaluation of Alexa Fluor 680-bombesin [7-14]NH_2_ peptide conjugate, a high-affinity fluorescent probe with high selectivity for the gastrin-releasing peptide receptor. Mol. Imaging.

[37] Bratanovic I.J., Zhang C., Zhang Z., Kuo H.T., Colpo N., Zeisler J., Merkens H., Uribe C., Lin K.S., Bénard F. (2022). A Radiotracer for Molecular Imaging and Therapy of Gastrin-Releasing Peptide Receptor-Positive Prostate Cancer. J. Nucl. Med..

[38] Mansi R., Minamimoto R., Mäcke H., Iagaru A.H. (2016). Bombesin-Targeted PET of Prostate Cancer. J. Nucl. Med..

[39] Carlucci G., Ananias H.J., Yu Z., Hoving H.D., Helfrich W., Dierckx R.A., Liu S., de Jong I.J., Elsinga P.H. (2013). Preclinical evaluation of a novel ¹¹¹In-labeled bombesin homodimer for improved imaging of GRPR-positive prostate cancer. Mol. Pharm..

[40] Van de Wiele C., Phonteyne P., Pauwels P., Goethals I., Van den Broecke R., Cocquyt V., Dierckx R.A. (2008). Gastrin-releasing peptide receptor imaging in human breast carcinoma versus immunohistochemistry. J. Nucl. Med..

[41] Kähkönen E., Jambor I., Kemppainen J., Lehtiö K., Grönroos T.J., Kuisma A., Luoto P., Sipilä H.J., Tolvanen T., Alanen K. (2013). In vivo imaging of prostate cancer using [^68^Ga]-labeled bombesin analog BAY86-7548. Clin. Cancer Res..

[42] Dimitrakopoulou-Strauss A., Hohenberger P., Pan L., Kasper B., Roumia S., Strauss L.G. (2012). Dynamic PET with FDG in patients with unresectable aggressive fibromatosis: Regression-based parametric images and correlation to the FDG kinetics based on a 2-tissue compartment model. Clin. Nucl. Med..

[43] Dimitrakopoulou-Strauss A., Hohenberger P., Haberkorn U., Mäcke H.R., Eisenhut M., Strauss L.G. (2007). ^68^Ga-Labeled Bombesin Studies in Patients with Gastrointestinal Stromal Tumors: Comparison with ^18^F-FDG. J. Nucl. Med..

[44] Duan H., Davidzon G.A., Moradi F., Liang T., Song H., Iagaru A. (2023). Modified PROMISE criteria for standardized interpretation of gastrin-releasing peptide receptor (GRPR)-targeted PET. Eur. J. Nucl. Med. Mol. Imaging.

[45] Koller L., Joksch M., Schwarzenböck S., Kurth J., Heuschkel M., Holzleitner N., Beck R., von Amsberg G., Wester H.J., Krause B.J. (2023). Preclinical Comparison of the ^64^Cu- and ^68^Ga-Labeled GRPR-Targeted Compounds RM2 and AMTG, as Well as First-in-Humans [^68^Ga]Ga-AMTG PET/CT. J. Nucl. Med..

[46] Yi X., Wang F., Qin W., Yang X., Yuan J. (2014). Near-infrared fluorescent probes in cancer imaging and therapy: An emerging field. Int. J. Nanomed..

[47] Wang L.G., Montaño A.R., Masillati A.M., Jones J.A., Barth C.W., Combs J.R., Kumarapeli S.U., Shams N.A., van den Berg N.S., Antaris A.L. (2024). Nerve Visualization using Phenoxazine-Based Near-Infrared Fluorophores to Guide Prostatectomy. Adv. Mater..

[48] Liu Z., Liu S., Niu G., Wang F., Liu S., Chen X. (2010). Optical imaging of integrin alphavbeta3 expression with near-infrared fluorescent RGD dimer with tetra(ethylene glycol) linkers. Mol. Imaging.

[49] Erspamer V., Melchiorri P., Sopranzi N. (1973). The action of bombesin on the kidney of the anaesthetized dog. Br. J. Pharmacol..

[50] Na H.B., Song I.C., Hyeon T. (2009). Inorganic Nanoparticles for MRI Contrast Agents. Adv. Mater..

[51] Yang C.Y., Hsiao J.K., Tai M.F., Chen S.T., Cheng H.Y., Wang J.L., Liu H.M. (2011). Direct labeling of hMSC with SPIO: The long-term influence on toxicity, chondrogenic differentiation capacity, and intracellular distribution. Mol. Imaging Biol..

[52] Liu G., Gao J., Ai H., Chen X. (2013). Applications and potential toxicity of magnetic iron oxide nanoparticles. Small.

[53] Singh N., Jenkins G.J., Asadi R., Doak S.H. (2010). Potential toxicity of superparamagnetic iron oxide nanoparticles (SPION). Nano Rev..

[54] Fesnak A.D., June C.H., Levine B.L. (2016). Engineered T cells: The promise and challenges of cancer immunotherapy. Nat. Rev. Cancer.

[55] Lu X., Horner J.W., Paul E., Shang X., Troncoso P., Deng P., Jiang S., Chang Q., Spring D.J., Sharma P. (2017). Effective combinatorial immunotherapy for castration-resistant prostate cancer. Nature.

[56] Qi X., Yang M., Ma L., Sauer M., Avella D., Kaifi J.T., Bryan J., Cheng K., Staveley-O’Carroll K.F., Kimchi E.T. (2020). Synergizing sunitinib and radiofrequency ablation to treat hepatocellular cancer by triggering the antitumor immune response. J. Immunother. Cancer.

