# Supplementary material for: Bimodal MRI/Fluorescence Nanoparticle Imaging Contrast Agent Targeting Prostate Cancer
Source: Nanomaterials (Basel). 2024 Jul 10;14(14):1177. doi: 10.3390/nano14141177 (PMC11279443; doi:10.3390/nano14141177)
Supplement: Supplementary file 1 [file nanomaterials-14-01177-s001.zip › nanomaterials-3076136-supplementary.pdf]

Supplementary Materials

Bimodal MRI/Fluorescence Nanoparticle Imaging Contrast Agent Targeting Prostate Cancer

Hang Xu [1,2,3,†], Ping Yu [4], Rajendra P. Bandari [1,3], Charles J. Smith [1,3,5], Michael R. Aro [1,3], Amolak Singh [1] and Lixin Ma [1,3,*]

[1] Department of Radiology, University of Missouri, Columbia, MO 65212, USA; xuhang@renji.com (H.X.); bandarir@health.missouri.edu (R.P.B.); smithcj@health.missouri.edu (C.J.S.); arom@health.missouri.edu (M.R.A.); singha@health.missouri.edu (A.S.)
[2] Department of Chemical Engineering Graduate Program, University of Missouri, Columbia, MO 65211, USA
[3] Harry S. Truman Veterans' Memorial Hospital, Columbia, MO 65201, USA
[4] Department of Physics and Astronomy, University of Missouri, Columbia, MO 65211, USA; pingyu@missouri.edu
[5] University of Missouri Research Reactor (MURR), University of Missouri, Columbia, MO 65211, USA
* Correspondence: mal@health.missouri.edu; Tel.: +1-(573)-814-6000 (ext. 53760); Fax: +1-(573)-814-6551
† Current address: State Key Laboratory of Systems Medicine for Cancer, Shanghai Cancer Institute, Shanghai Key Laboratory for Cancer Systems Regulation and Clinical Translation (CSRCT-SHANGHAI), Renji Hospital Affiliated to Shanghai Jiao Tong University School of Medicine, Shanghai 200127, China.

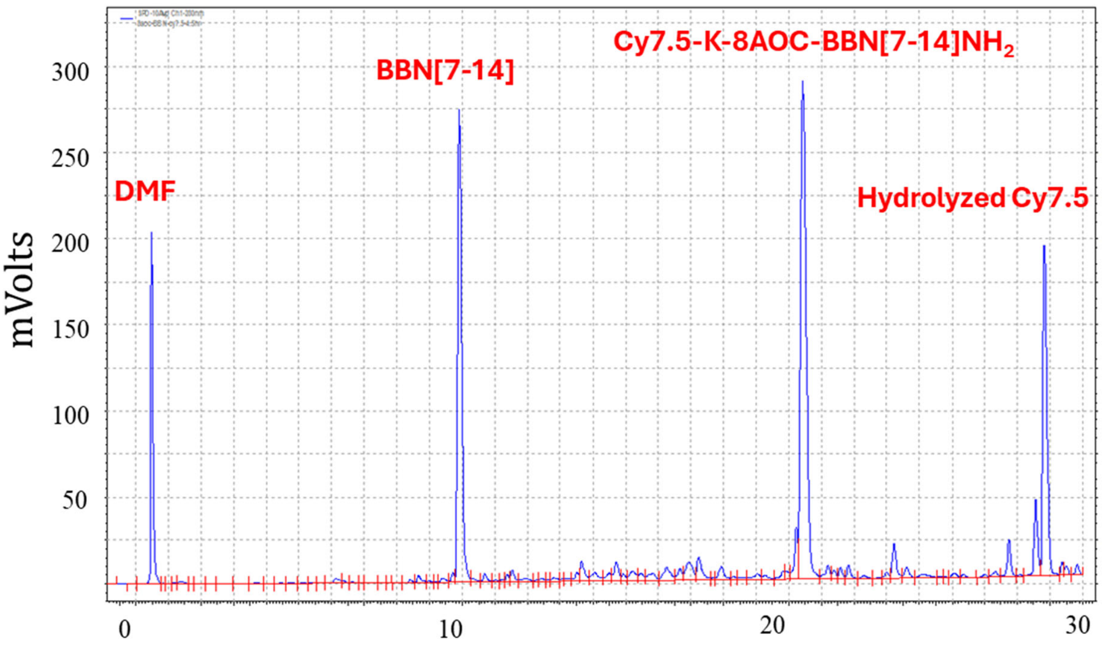

Figure S1. HPLC profile for the purification of Cy7.5-K-8AOC-BBN[7-14]NH_2.

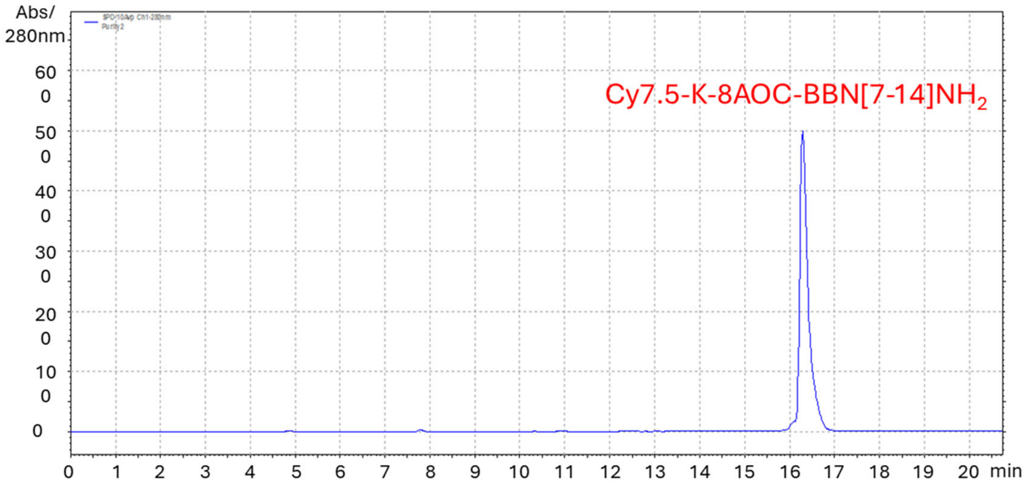

Figure S2. Purity determination with HPLC of the Cy7.5-K-8AOC-BBN[7-14]NH2. The Purity is over 95%.

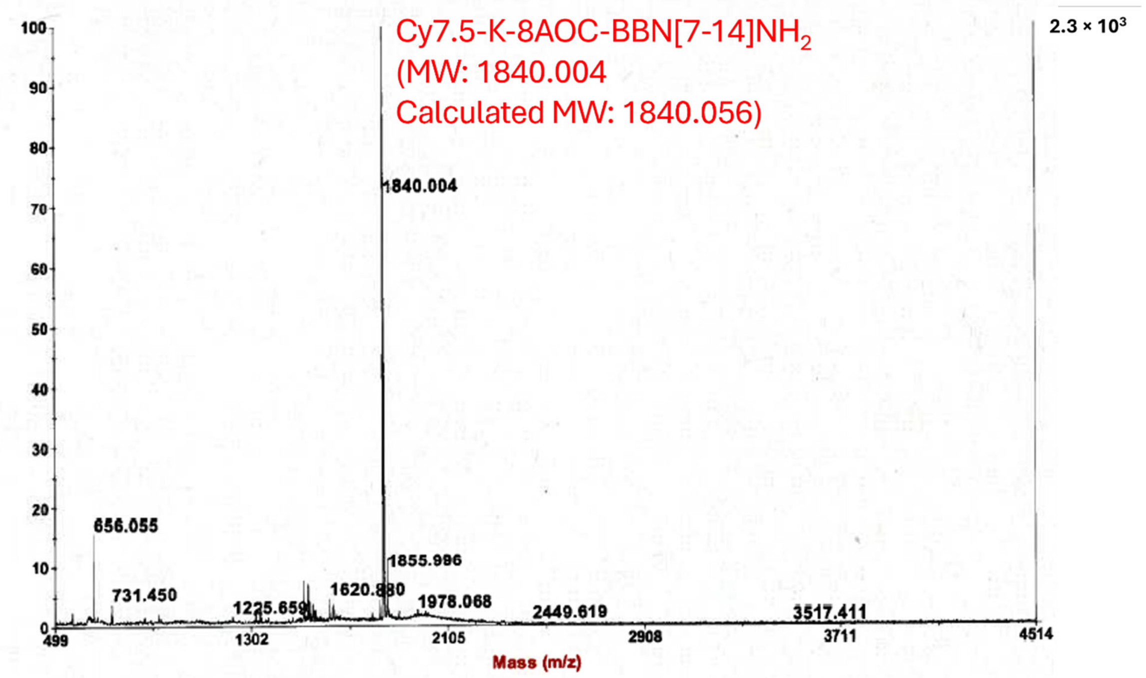

Figure S3. Mass spectrum of the Cy7.5-BBN[7-14] or K(Cy7.5)-8AOC-BBN[7-14]NH2. The molecular weight (MW) in the figure is 1840.0 Da, matching the theoretical MW of 1840.1.

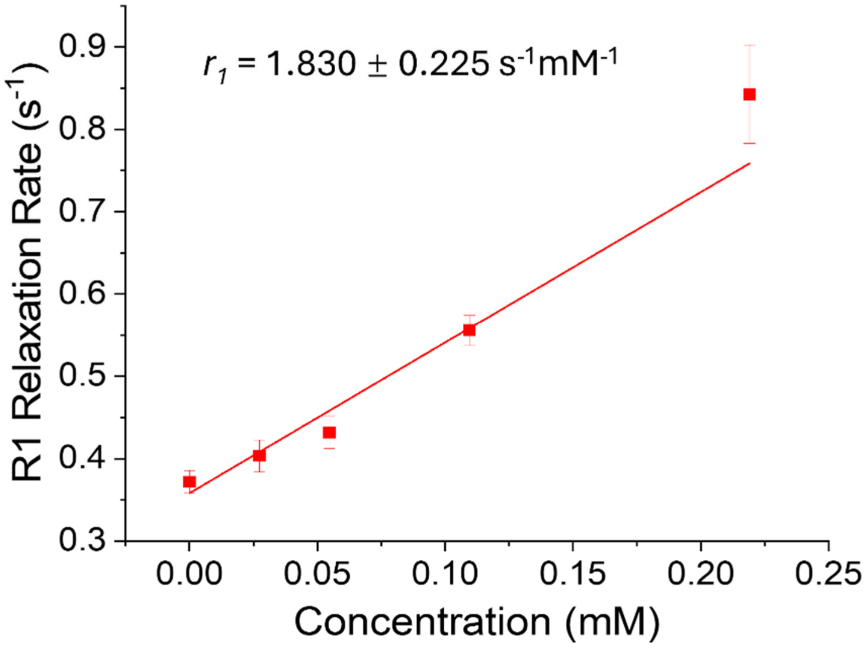

Figure S4. Linear fitting of R_1 relaxation rates against concentration of USPIO(Cy7.5)-BBN. The relaxivity was determined to be r_1 = 1.830 ± 0.225 s^{-1} mM^{-1} for USPIO(Cy7.5)-BBN at 7T MRI and room temperature.

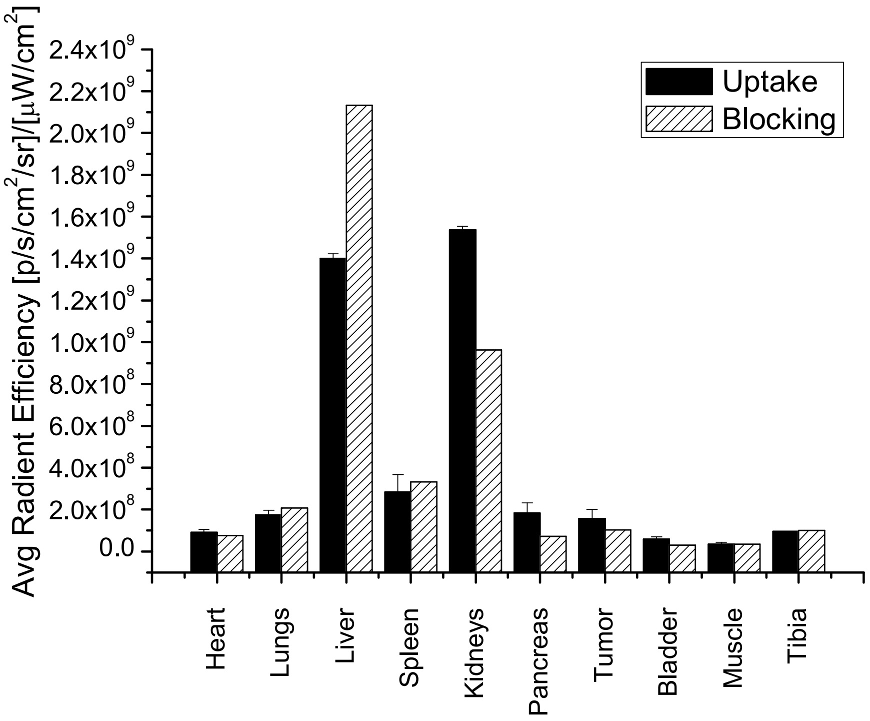

Figure S5. Bio-distribution of USPIO(Cy7.5)-BBN in SCID mice bearing PC-3 xenografts determined by ex vivo NIRF imaging analysis.
